# Interleukin-22 From Type 3 Innate Lymphoid Cells Aggravates Lupus Nephritis by Promoting Macrophage Infiltration in Lupus-Prone Mice

**DOI:** 10.3389/fimmu.2021.584414

**Published:** 2021-02-26

**Authors:** Lingzhen Hu, Jingyi Hu, Liheng Chen, Yi Zhang, Qingqing Wang, Xuyan Yang

**Affiliations:** ^1^ Department of Rheumatology, Second Affiliated Hospital, College of Medicine, Zhejiang University, Hangzhou, China; ^2^ Laboratory of Immunology, College of Medicine, Institute of Basic Medical Sciences, Zhejiang University, Hangzhou, China; ^3^ Department of Rheumatology, Hangzhou Dingqiao Hospital, Hangzhou, China

**Keywords:** systemic lupus erythematosus, lupus nephritis, interleukin-22, interleukin-22 receptor, macrophage

## Abstract

Lupus nephritis (LN) is one of the most severe manifestations of systemic lupus erythematosus (SLE). Our previous studies demonstrated increased serum and renal Interleukin (IL)-22 in LN patients and MRL/lpr mice. This study investigated the role of IL-22 and its mechanism in LN. Here, we found that IL-22 was mainly produced by type 3 innate lymphoid cells (ILC3) in kidney of MRL/lpr mice. The systemic illness and local renal lesion were significantly alleviated in IL-22 or IL-22R gene knockout (KO) mice (IL-22 KO or IL-22R KO MRL/lpr mice) than control mice (MRL/lpr mice). IL-22 KO or IL-22R KO MRL/lpr mice had significantly slighter infiltration of macrophage in kidney than MRL/lpr mice. Consistently, by RNA-Seq, the expression of (CC motif) ligand 2 (CCL2) and (CXC motif) ligand 10 (CXCL10) was decreased in kidney of KO mice compared with control mice. By immunoblotting, significantly increased levels of STAT3 phosphorylation were found in the kidney of control mice compared to KO mice. *In vitro*, primary kidney epithelial cells from control mouse stimulated with recombinant IL-22 (rIL-22) expressed higher levels of CCL2, CXCL10, and phosphorylated STAT3. At the same time, when primary kidney epithelial cells were treated with rIL-22, transwell assay demonstrated their supernatant recruited more macrophages. In human kidney epithelial cell line (HK2) cells, when treated with rIL-22, we observed similar results with primary mouse kidney epithelial cells. Moreover, when cells were stimulated with rIL-22 following pre-treatment with STAT3 pathway inhibitor, the expression of CCL2 and CXCL10 were significantly reversed. Our findings demonstrate that IL-22 binding to IL-22R in kidney epithelial cells activated the STAT3 signaling pathway, enhanced the chemokine secretion and then promoted macrophage infiltration to the kidney of MRL/lpr mice, thus aggravated LN in lupus-prone mice. These findings indicate that IL-22 may play a pathogenic role in LN and may provide a promising novel therapeutic target for LN.

## Introduction

Systemic lupus erythematosus (SLE) is a chronic inflammatory disease, resulting from auto-immune dysfunction, and presenting with immune complex-mediated lesions in diverse organ (1). Lupus nephritis (LN) is one of the most severe manifestations of SLE (2). Despite the improvement of therapeutic approaches in recent years, 12-month complete renal response rates are only 10%–40% (3), and for all LN patients, the cumulative incidence of end-stage renal disease (ESRD) at 10 years after the diagnosis of LN was 10.1% (4). Therefore, a novel therapeutic target for LN is pressing and timely.

The interleukin (IL)-22 belongs to the IL-10 cytokine family (5). IL-22 is primarily produced by T cell subsets and innate lymphoid cells (ILCs) (6) but preferentially act on non-hematopoietic cells, particularly epithelial cells because IL-22 receptor (IL-22R) is restricted to the epithelial cells of tissues, including the skin, intestine, liver, lung and kidney (7). IL-22 mediates its effects *via* the IL-22-IL-22R complex and subsequent Janus kinase–signal transducer and activators of transcription (JAK–STAT) signaling pathway (5). Accumulated evidence has indicated that IL-22 plays a pathogenic or protective role in different autoimmune diseases (8–10). We previously found that IL-22 was increased in serum and renal tissue of both LN patients and MRL/lpr mice (11), and that elevated urinary IL-22 binding protein(IL-22BP) in LN patients were associated active renal disease (12). Our further experiments demonstrated that MRL/lpr mice treated with anti-IL-22 monoclonal antibody (mAb) had substantial improvement of renal function and less renal injury (11).

Based on observations above, we hypothesized that IL-22 plays a central role in the pathogenesis of LN. We respectively knocked out IL-22 and IL-22R gene of MRL/lpr mice to investigate the role of IL-22 and its mechanism in LN.

## Methods

### Mice

MRL/lpr female mice were used as the model of lupus. They were obtained from Shanghai Slac Laboratory Animal CO. LTD (Shanghai, China). IL-22 knockout mice deleted of IL-22 exons 1 through 4 (NM_016971) were purchased from Mutant Mouse Resource and Research Centers (MMRRC, USA). IL-22 receptor knockout mice were purchased from Nanjing Biomedical Research Institute of Nanjing University (Nanjing, China). All mice were housed in a specific pathogen free condition in the animal facility at School of Medicine, Zhejiang University, China. IL-22 knockout and IL-22R knockout mice were bred to MRL/lpr mice (designed as control) in our colony and backcrossed for at least 10 generations to generate IL-22^-/-^ MRL/lpr (designed as IL-22 KO) and IL-22R^-/-^ MRL/lpr (designed as IL-22R KO). All animal experiments were performed according to the protocol approved by the Ethics Committee of the Second Affiliated Hospital, College of Medicine, Zhejiang University in compliance with institutional guidelines.

### Patients

Ten LN patients were recruited from March 2017 to December 2018 at the Department of Nephrology and Rheumatology of the Second Affiliated Hospital, College of Medicine, Zhejiang University. All patients fulfilled the American College of Rheumatology (ACR) diagnostic criteria of SLE (13) and was defined by renal biopsy. Three normal renal tissues from para-carcinoma tissues as healthy controls (HCs) were confirmed by light microscope examination. Renal biopsy was handled under ultrasound local isolation. Renal tissue was extracted for immunohistochemical assessment. The study protocol was approved by the Ethics Committee of the Hospital and was conducted in accordance with the 1989 Declaration of Helsinki.

### Primary Mouse Kidney Epithelial Cells

Freshly isolated kidneys were placed in ice-cold DMEM mixed with Hams F12 (1:1 ratio; Life Technologies, Grand Island, NY) on a 60 mm dish. The kidney capsule was removed by peeling with forceps, and the kidney was sliced coronally and homogenized by mincing into 1–2 mm^3^ pieces. The homogenized kidney tissue pieces were resuspended and mixed in 10 ml of collagenase type IV for 30 min at 37°C to obtain single-cell suspensions. After digestion, the cell suspension was filtered through 70-μm cell strainers. The filtered cell suspensions were centrifuged at 300*g* for 5 min and incubated with ACK lysing buffer (Beyotime Biotechnology, China) to remove red blood cells. Then, the pellet was washed with DMEM/F12 medium with 10% FBS twice and passed through a 40-μm cell strainer. After filtering, cells were generated in DMEM/F12 medium with 10% FBS on a 60 mm dish. Then, medium was replaced with fresh DMEM/F12 medium with 10% FBS 6 h later.

### Cell Culture and IL-22 Treatment *In Vitro*

The HK-2 cells were obtained from the Cell Bank of the Chinese Academy of Sciences (Shanghai, China) and maintained in DMEM/F12 medium with 10% FBS. After FBS starvation in DMEM/F12, cells at 80% confluence were stimulated with recombinant IL-22 (rIL-22) (100 ng/ml) (Peprotech). To inhibit the STAT3 signaling pathways, C188-9 (MCE) was added 24 h before stimulation with IL-22.

### Isolation of Spleen and Kidney Cells

Freshly isolated spleens were placed in ice-cold DMEM (Life Technologies, Grand Island, NY) on a 60 mm dish. Spleens were grinded and passed through a 40-μm cell strainer. The pellet was incubated with ACK lysing buffer (Beyotime Biotechnology) for 2 min at room temperature. The renal single-cell suspensions were obtained according to Chong’s protocol (14). Then, the cells were prepared for staining and flow cytometry analysis.

### Flow Cytometry

Single-cell suspensions obtained from the blood, spleen and kidney were stimulated at 37°C for 6 h at 5% CO2 in the presence of 50 ng/ml phorbol myristate acetate (Sigma-Aldrich), 1 μg/ml ionomycin (Sigma-Aldrich), and Brefeldin A Solution (BD Bioscience, USA). After incubation, the cells were blocked with anti-CD16/CD32 antibodies (Biolegend, USA) and then stained with the indicated antibodies for 20 min at 4°C. After fixation and permeabilization with Perm/Wash solution (BD Pharmingen, USA), the cells were stained with allophycocyanin-conjugated anti-IL22 monoclonal antibodies (Biolegend, USA) in the dark at 4°C for 30 min. Then, the cells were washed with Perm/Wash solution once and resuspended. Isotype controls were utilized to enable accurate compensation and to confirm the antibody specificity. The stained cells were analyzed by FCM using ACEA NovoCyte™ cytometer (ACEA Biosciences, USA) and analyzed with NovoExpress software (ACEA Biosciences, USA) and FlowJo software (Tree Star Inc, Ashland, OR, USA). Refer to the antibody table (**Supplemental Table 2**) for a complete list of antibodies.

### Assessment of Proteinuria and Renal Function

Blood urea nitrogen (BUN) was determined by the Quantichrom DIUR 500 kit (BioAssay Systems, Hayward, CA). Serum and urinary creatinine were measured by the QuantiChrom Creatinine Assay Kit (BioAssay Systems). The ratio of albumin to creatinine in urine was measured using an ELISA kit (Exocell).

### Gross Pathology

Skin lesions were scored at age of 24 weeks. We scored the skin lesions by gross pathology using a grade of 0–6 (0 = none; 1 = mild, < 2 cm; 2 = severe, ≥2 cm, scored the snout, ears, and intrascapular separately and added). Lymphadenopathy (cervical, brachial, and inguinal) was graded as 0–4 (0=none; 1=small, one site; 2=moderate, two sites; 3=large, three sites; and 4=very large, four sites or more) and splenomegaly was analyzed by spleen weight upon euthanasia.

### Renal Histopathology

Kidneys were fixed in 10% neutral buffered formalin, embedded in paraffin, sectioned (4 μm), and stained with hematoxylin and eosin (H& E), or periodic acid-Schiff (PAS). To score kidney pathology we evaluated glomerular pathology as previously described (11).

### Immunohistochemistry

Paraffin-embedded Kidney sections (5 μm thick) were deparaffinised and boiled for 10 min in sodium citrate buffer (10 mM, pH 6.0). The sections were depleted of endogenous peroxidase activity by adding methanolic H_2_O_2_ for 10 min and blocked with normal serum for 30 min. After overnight incubation at 4°C with polyclonal antibodies against CD68 (Abcam, USA) or pSTAT3 (CST, USA), the samples were incubated for 30 min with the secondary antibody, biotinylated anti-rabbit IgG (CD68), or biotinylated anti-mouse IgG(pSTAT3), and incubated with streptavidin–peroxidase complex (Vector) for 1 h, followed by incubation with 3,3’-diaminobenzidine (Dako) for 5 min. The sections were counterstained with haematoxylin. The samples were photographed using an Olympus photomicroscope.

### Immunofluorescence

Immunofluorescence was carried out using standard protocol on kidney sections. Briefly, heat mediated antigen retrieval with Tris/EDTA buffer pH 6.0 was performed before commencing with staining protocol. After 30 min in BSA (G5001, Servicebio), slides were incubated overnight with specific primary antibody, rabbit anti-mouse IgG (Abcam, USA) and rat anti-mouse C3 (Santa Cruz, USA) at 4°C overnight, followed by FITC-conjugated secondary antibody (Servicebio, China). Nuclei were counterstained with 4’,6-diamidino-2-phenylindole (DAPI). The samples were photographed using NIKON ECLIPSE C1.

### ELISA

We measured serum total IgG, ds-DNA antibody, C3 (Abcam) by ELISA kits according to the manufacturer’s protocol. Briefly, standard or sample was added into each well and incubated. The supernatant was removed, and biotin-antibody was added and incubated. After three times wash with PBS, HRP-avidin was added into each well and incubated, followed by incubation with 3,3’,5,5’ tetramethylbenzidine (TMB) substrate for 20 min in dark. The reaction was terminated by adding stop solution, and the optical density was measured within 30 min using a microplate reader at 450 nm. All samples were assayed in triplicate.

### Quantitative Real-Time PCR

The total RNA was isolated from the cells using TRIzol reagent (Takara) according to the manufacturer’s directions, and single-strand cDNA was generated from the total RNA and reverse transcriptase (Toyobo). The SYBR Green master Rox (Roche) was used for the quantitative real-time reverse transcription–PCR analysis according to the manufacturer’s protocol. The relative gene quantification was done using the 2-^ΔΔ^Ct method following normalization to internal control glyceraldehyde-3-phosphate dehydrogenase (GAPDH). The Primers used for qPCR were synthesized by Tsingke Biotech Co. Ltd (Beijing, China). Refer to the primer table (**Supplemental Table 1**) for a complete list of primers in this study.

### Transwell Chemotaxis Assay

Primary bone marrow derived macrophages (BMDMs) were obtained according to Chong’s protocol (14). 1 × 10^5^ BMDMs were added to the upper chambers of a 5-μm pore polycarbonate Transwell filter (Corning). The upper and lower chambers were incubated for 30 min at 37°C in macrophage starve medium. The medium in the lower chamber was replaced with supernatant from mouse kidney epithelial cells treated or not with rIL-22. After 24 h, transmigrated cells were fixed and stained using the Crystal Violet Staining Solution (Beyotime, China). For each filter, 10 random images were acquired using OLYMPUS IX53 microscope.

### Immunoblotting

Kidney tissues were homogenized in RIPA buffer (50 mM TriszHCl [pH 7.4], 150 mM NaCl, 1% NP40, 1 mM PMSF, 1 mM NaF, 20 mM Na4P2O7, 2 mMNa3VO4, and 13 protease inhibitor cocktail [Roche Applied Science, Indianapolis, IN]), and equal protein (30 mg) was resolved by PAGE. Proteins from kidneys or HK-2 cells were transferred onto nitrocellulose membrane, and immunoblotting was performed with mouse polyclonal anti-pSTAT3, STAT3, GAPDH, and rabbit polyclonal anti-p38, p-p38 (Cell Signaling Technology) antibodies. Images were captured using the ChemiDocMP Imaging System (Bio-Rad, Hercules, CA).

### RNA-Seq

Total RNA from kidneys was extracted using TRIzol (Takara). Preparation of the library and transcriptomic sequencing were carried out using the Illumina HiSeq ×Ten (Novogene Bioinformatics Technology). Mapping of 100-bp paired-end reads to genes was done using HTSeq software (version 0.6.0), and fragments per kilobase of transcript per million fragments mapped (FPKM) were also analyzed. The sequencing data was submitted to Sequence Read Archive (SRA) of NCBI and the SRA accession is PRJNA648341.

### Statistical Analysis

Data was analyzed using Graph Pad Prism 7, and presented as mean ± standard deviation. For experiments with only two groups, significance was determined by a Student’s t test or the Mann-Whitney U-test according to whether the distribution is normal or not. Significance for >2 groups was determined by one-way analysis of variance (ANOVA). Post hoc least significant difference (LSD) tests, in which all groups were tested against a control group as a reference, were performed if the results of the initial analysis of variance were significant. p-values <0.05 were considered significant and marked with one asterisk, while the p-values of <0.01 or <0.001 were marked with two or three asterisks.

## Results

### IL-22 Was Mainly Expressed by ILC3s in Kidney of Lupus-Prone Mice

**Figure 1:**
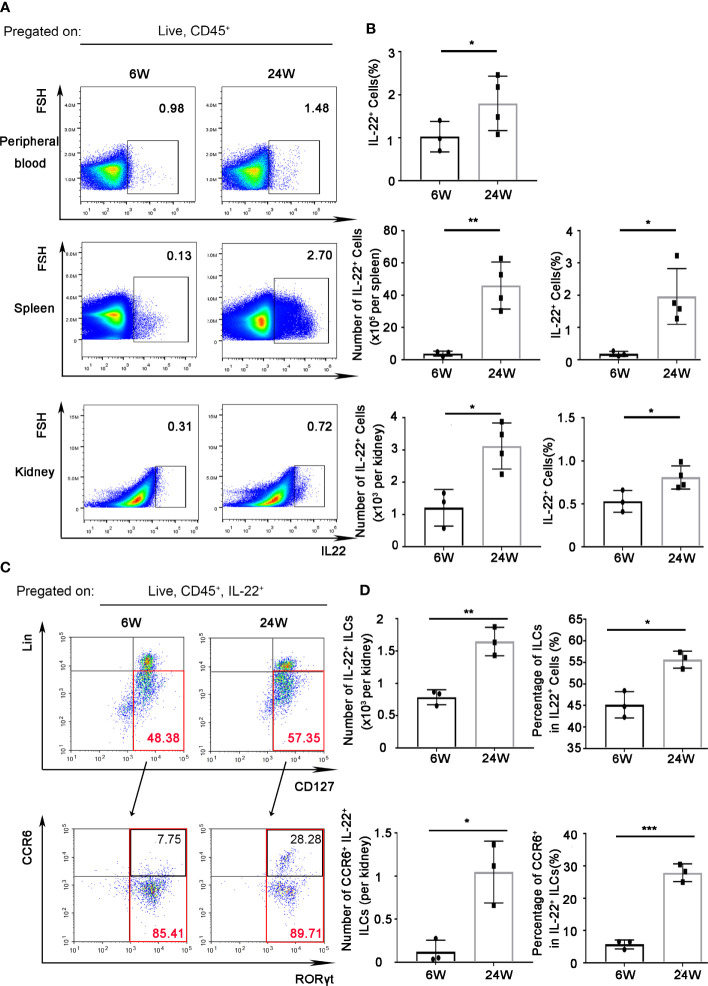
IL-22 was mainly expressed by ILC3s in kidney of lupus-prone mice. IL-22^+^ cells in peripheral blood, spleen, kidney from 6-weeks or 24-weeks-old MRL/Fas Mice were analyzed by flow cytometry. **(A)** The gating strategy for detecting IL-22^+^ cells in peripheral blood, spleen, and kidney. **(B)** The quantitative analysis of the percentage and amount of IL-22^+^ cells in peripheral blood, spleen, and kidney. **(C, D)** The gating strategy for detecting IL-22^+^ cell subsets and analysis of IL-22^+^ ILCs (Lin^-^CD127^+^), IL-22^+^ILC3s (Lin^-^CD127^+^RORγt^+^), and CCR6^+^ IL-22^+^ ILC3s in kidney. Data were expressed as mean ± SD, and are representative of three independent experiments. T-test was used for comparison between groups (*P < 0.05, **P < 0.01, ***P < 0.001). ILCs, innate lymphoid cells.

As we had found increased level of IL-22 in serum and renal tissue from LN patients (11), we determined to explore the expression of IL-22 in MRL/lpr mice. In peripheral blood, the percentage of IL-22^+^ cells in leukocytes increased significantly in 24-weeks-old MRL/lpr mice compared to 6-weeks-old mice (**Figures 1A, B**). In spleen, total amount and the percentage of IL-22^+^ cells in leukocytes were both significantly increased in 24-weeks-old MRL/lpr mice (**Figures 1A, B**).

In kidney, total amount and the percentage of IL-22^+^ cells in leukocytes also increased in 24-weeks-old MRL/lpr mice compared to 6-weeks-old MRL/lpr mice (**Figures 1A, B**). At the same time, we found that the majority (nearly 60%) of IL-22^+^ cells in kidney of 24 weeks-old mice were IL-22^+^ innate lymphoid cells (ILCs, Lin^-^CD127^+^) (**Figure 1C**). Moreover, the amount of IL-22^+^ ILCs increased with the development of age-related lupus nephritis (**Figure 1D**), while the absolute number of IL-22^+^ T cells (IL-22^+^CD3^+^) in the kidneys showed no significant difference (**Supplementary Figure 1**). And IL-22^+^ ILCs were almost all (nearly 90%) from ILC3 (Lin^-^CD127^+^RORγt^+^) subgroup (**Figure 1C**). Unexpectedly, we found the percentage of CCR6^+^ IL-22^+^ ILC3s that can secrete IL-17 cytokine in 24-weeks-old MRL/lpr mice also significantly increased compared to 6-weeks-old mice (**Figures 1C, D**).

### IL-22 Shortened Survival and Promoted Systemic Illness in Lupus-Prone Mice

**Figure 2:**
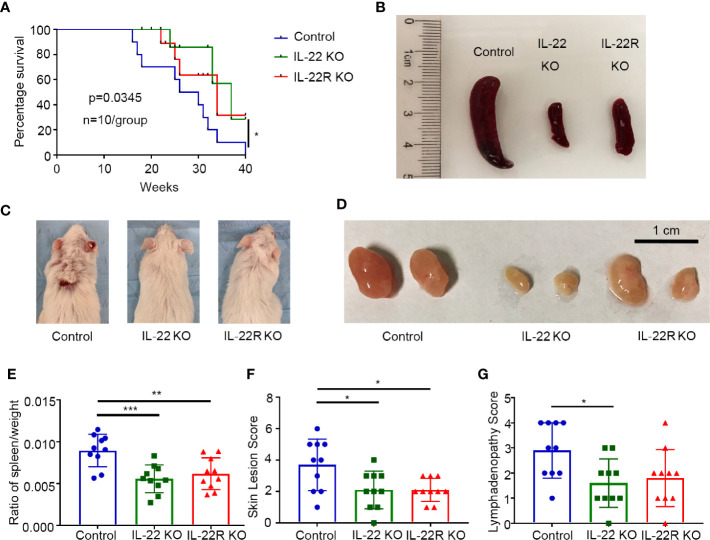
IL-22 shortened survival and promoted systemic illness in lupus-prone mice. **(A)** Survival curve of control, IL-22 KO, and IL-22R KO mice (n=10/group) over 10 months. **(B–D)** Representative images of spleen, skin, and lymph node from control, IL-22 KO, and IL-22R KO mice (24 weeks old mice). **(E)** The ratio of the spleen/weight, **(F)** skin lesion score and **(G)** lymphadenopathy score were compared between 24-weks-old control, IL-22 KO, and IL22-R KO mice. Skin lesions were scored by gross pathology using a grade of 0–6 (0 = none; 1 = mild, < 2 cm; 2 = severe, ≥2 cm, scored the snout, ears, and intrascapular separately and added). Lymphadenopathy (cervical, brachial, and inguinal) was graded as 0–4 (0=none; 1=small, one site; 2=moderate, two sites; 3=large, three sites; and 4=very large, four sites or more). Data were expressed as mean ± SD, and were representative of three independent experiments. One way ANOVA was used for comparison among all groups (*P < 0.05, **P < 0.01, ***P < 0.001).

To further confirm the role of IL-22 in the pathogenesis of LN, we performed experiments on IL-22 KO or IL-22R KO mice. IL-22 KO or IL-22R KO MRL/lpr mice survived longer than control mice (**Figure 2A**). These control mice underwent substantial proteinuria, oliguria and anuria before death (not shown).

We also detected the spleen/weight ratio, skin lesions and lymphadenopathy in 24-weeks-old mice. Gross specimens showed control mice had much severer splenomegaly than IL-22 KO or IL-22R KO mice (**Figure 2B**), and consistently the spleen/weight ratio in MRL/lpr mice was significantly increased compared to IL-22 KO or IL-22R KO mice (**Figure 2E**). The score of skin lesions in control mice was significantly higher than that in IL-22 KO or IL-22R KO mice (**Figures 2C, F**). The score of lymphadenopathy in control mice was significantly increased compared to IL-22 KO mice, and also higher than that in IL-22R KO mice though without significant difference (**Figures 2D, G**).

### IL-22 or IL-22R Deficiency Ameliorated Renal Injury in Lupus-Prone Mice

**Figure 3:**
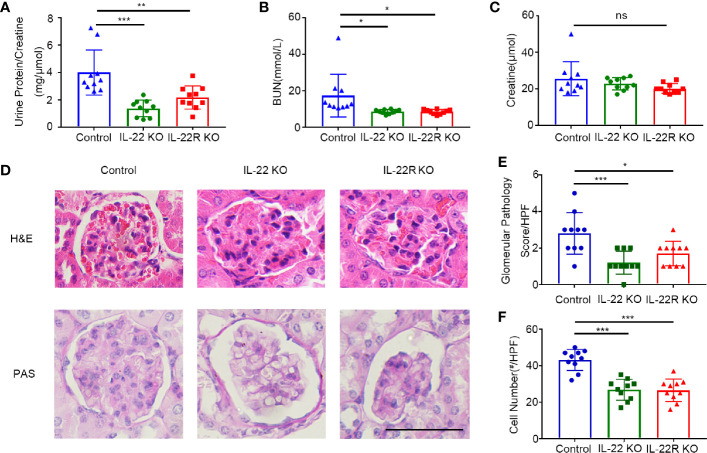
IL-22 or IL-22R deficiency ameliorated renal injury in lupus-prone mice. Renal function as **(A)** urine protein/creatinine ratio, **(B)** serum BUN, **(C)** serum creatinine of 24-weeks-old control and IL-22 KO, IL-22R KO mice. **(D)** Representative images of kidney biopsies stained in H&E and PAS from control, IL-22 KO, and IL-22R KO mice (24 weeks, scale bar, 50 μm). The glomerular pathology score **(E)** and cell numbers per glomerulus **(F)** were analyzed and counted in the PAS stained slides from 24-weeks-old control, IL-22 KO and IL-22R KO mice. Data were expressed as mean ± SD, and are representative of three independent experiments. One way ANOVA was used for comparison among all groups (*P < 0.05, **P < 0.01, ***P < 0.001).

We next explored the effect of IL-22 KO or IL-22R KO on LN. Proteinuria, renal function and magnitude of renal pathology were examined in 24-weeks-old mice. The ratio of albuminuria/creatinine in IL-22 KO mice was significantly decreased, compared with control mice (**Figure 3A**), and serum BUN levels in both IL-22 KO and IL-22R KO mice were significantly lower than control mice (**Figure 3B**). The serum creatinine levels in KO mice also decreased, though without statistical significance (**Figure 3C**).

The pathological changes in kidneys of lupus-prone mice were analyzed morphologically as shown by H&E staining. There was significantly less renal injury in IL-22 KO or IL-22R KO mice than those in control mice (**Figure 3D**). At 24 weeks of age, more glomerular cellularity, collapse of capillary lumina, and thicker basement membranes were observed in control mice as shown by Periodic Acid-Schiff (PAS) staining (**Figure 3D**). The glomerular pathology score (**Figure 3E**) and the number of cells per glomerulus (**Figure 3F**) were significantly lower in IL-22 KO or IL-22R KO mice than those in control mice. Thus, deleting IL-22 or IL-22R gene ameliorated renal injury in lupus-prone Mice

### IL-22 or IL-22R Deficiency Altered the Levels of Autoantibody and Complement in Lupus-Prone Mice

**Figure 4:**
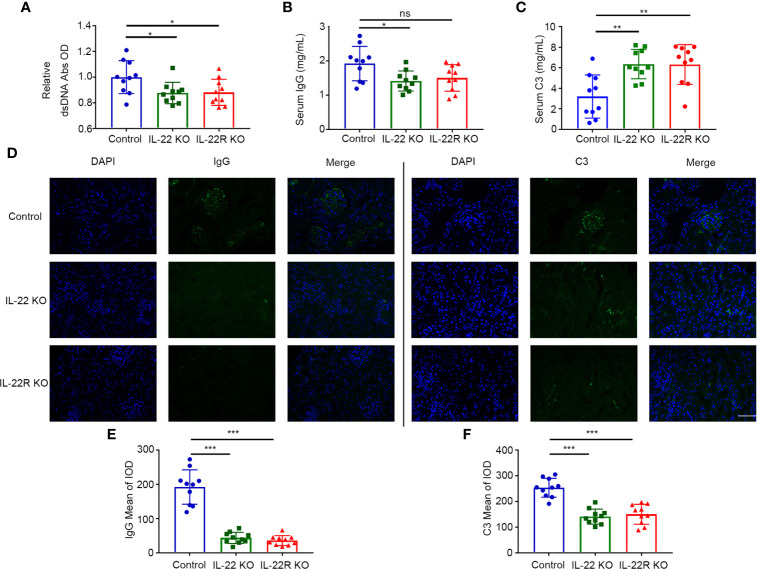
IL-22 or IL-22R deficiency altered the levels of autoantibody and complement in lupus-prone mice. **(A–C)** The level of serum dsDNA Abs, total IgG and C3 were measured by ELISA in 24-weeks-old control, IL-22 KO and IL-22R KO mice. **(D)** Representative photos of IgG and C3 deposition in the glomeruli of 24-weeks-old control, IL-22 KO and IL-22R (scale bar, 50 μm). **(E, F)** Quantification of the mean IOD of IgG and C3 per glomerular cross-section in all groups of mice. Data were expressed as mean ± SD, and are representative of three independent experiments. One way ANOVA was used for comparison among all groups (*P < 0.05, **P < 0.01, ***P < 0.001).

We examined the effect of IL-22 or IL-22R deficiency on serum anti-dsDNA autoantibody (Ab) production. The levels of dsDNA Ab were significantly lower in 24-weeks-old IL-22 or IL-22R KO mice than control mice of the same age (**Figure 4A**). We also detected serum total IgG and C3. The levels of serum total IgG were lower in IL-22 KO mice than control mice (**Figure 4B**). Conversely, the levels of serum C3 were significantly higher in IL-22 or IL-22R KO mice than control mice (**Figure 4C**). We also detected the IgG and C3 immune deposition in the glomeruli of 24-weeks-old mice by immunofluorescence (IF). The IgG and C3 immune deposition in the glomeruli of IL-22 or IL-22R KO mice was significantly alleviated (**Figures 4D–F**).

### IL-22 or IL-22R Deficiency Reduced Intrarenal Macrophages by Decreasing the Expression of CCL2 and CXCL10 in Lupus-Prone Mice

**Figure 5:**
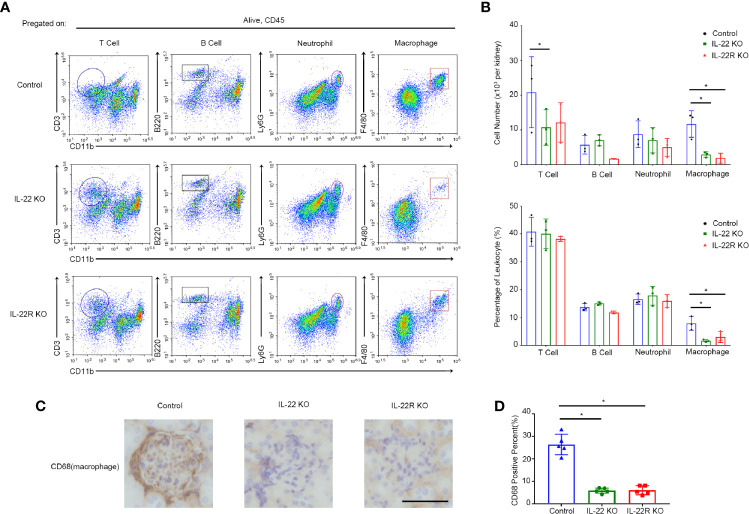
IL-22 or IL-22R deficiency reduced intrarenal macrophages in lupus-prone mice. **(A)** Kidney cells from 24-weeks-old IL-22 KO, IL-22R KO and control Mice were analyzed by flow cytometry for the percentage of different inflammatory cell subsets, including T cells, B cells, neutrophils and macrophages. **(B)** Quantitative analysis of the amount and the percentage of different cell subsets per kidney in three groups. **(C)** Representative photos of macrophages (marked by CD68) in control IL-22 KO and IL-22R KO mice glomeruli at 24 weeks of age (scale bar, 50 μm). **(D)** Quantitative analysis of the percentage of CD68 positive cells in the kidneys of mice from three groups. Data were expressed as mean ± SD, and are representative of three independent experiments. One way ANOVA was used for comparison among all groups (*P < 0.05).

By flow cytometry, we detected the expression of these inflammatory cells, including T cells, B cells, neutrophils and macrophages, in the kidney of lupus-prone Mice. The number and the percentege of infiltrating macrophages in the kidneys of IL-22 or IL-22R KO mice was significantly decreased compared with control mice, but there was no significant difference in the number and the percentage of B cells and neutrophils among three groups (**Figures 5A, B**). Though the number of T cells was higher than IL-22 KO mice, the percentage of them showed no difference (**Figure 5B**). Immunohistochemistry also showed less intrarenal macrophages in IL-22 or IL-22R KO mice than control mice (**Figures 5C, D**). These outcomes indicate that IL-22 or IL-22R deficiency reduced macrophages infiltration in lupus-prone mice.

**Figure 6:**
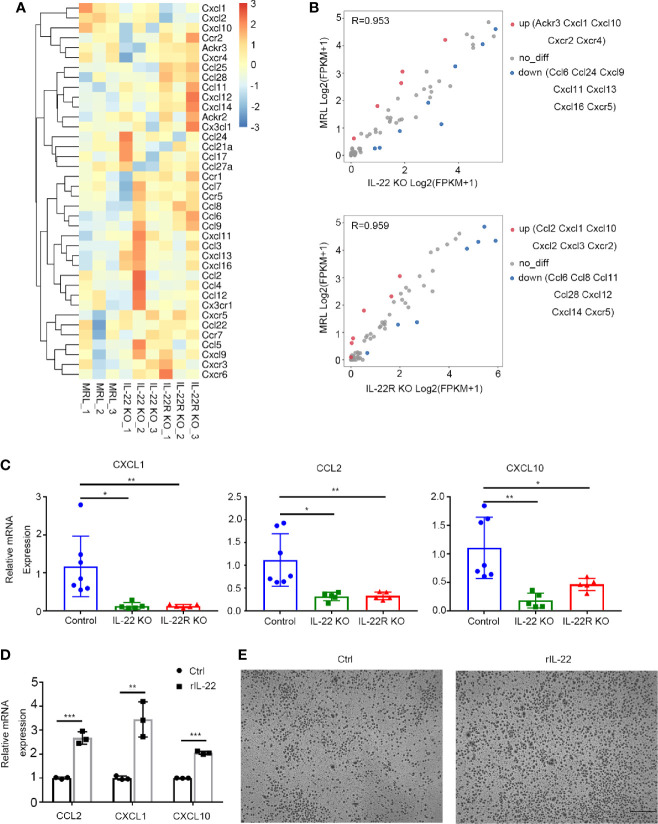
IL-22 affected the expression of chemokines *in vivo* and *in vitro*. **(A)** Gene expression profile of chemokines and their receptors in kidneys from control, IL-22 KO and IL-22R KO mice. (n = 3 independent experiments). For the gene expression profiles, the colored key shows fold change in gene expression. **(B)** Scatter plot of differential expression of chemokines and their receptors in kidneys from control, IL-22 KO and IL-22R KO mice. (n = 3 independent experiments, P value < 0.05 and fold change> 2). **(C)** Gene expression level of intrarenal chemokine was analyzed by quantitative reverse transcription PCR (qRT-PCR) in kidney of mice at 24 weeks of age. Values are normalized to Actin transcripts and expressed as relative ratio. **(D)** Gene expression level of chemokines in primary kidney epithelial cells from control mice treated with or without rIL-22 (100 ng/ml, 60 min) by qRT-PCR. **(E)** Representative photos of macrophages recruited by supernatant from primary kidney epithelial cells stimulated with or without rIL-22 for 24 h (scale bar, 500 μm). Data were expressed as mean ± SD, and are representative of three independent experiments. One way ANOVA was used for comparison among three groups, T-test was used for comparison between two groups (*P < 0.05, **P < 0.01, ***P < 0.001).

Then, we detected gene expression profile of intrarenal chemokines and chemokine receptors (**Figure 6A**). Lower expression of chemokine (C-X-C motif) ligand 1 (CXCL1) and IFNγ-induced protein-10 (CXCL10) were found in kidney of IL-22 or IL-22R KO compared with control mice (**Figure 6B**). Quantitative reverse transcription PCR (qRT-PCR) further confirmed that deficiency of IL-22 or IL-22R resulted in decreased levels of CXCL1, and monocyte chemo attractant protein-1 (MCP-1, CCL2) and CXCL10 (**Figure 6C**).

*In vitro*, we isolated the mouse kidney epithelial cells from MRL/lpr mice, and then stimulated it with 100 ng/ml recombined IL-22(rIL-22) for 60 min. It was shown that the relative mRNA expression of CCL2, CXCL1 and CXCL10 were significantly increased (**Figure 6D**). And the supernatant from cells stimulated with rIL-22 transferred to the lower chambers for 24 h attracted more macrophages by transwell chemotaxis assay (**Figure 6E**), suggesting that IL-22 may recruit macrophages by upregulating the expression of CCL2, CXCL1, and CXCL10 in kidney epithelial cells.

### IL-22 Promoted Kidney Epithelial Cells to Express CCL2 and CXCL10 by Activating the STAT3 Pathway

**Figure 7:**
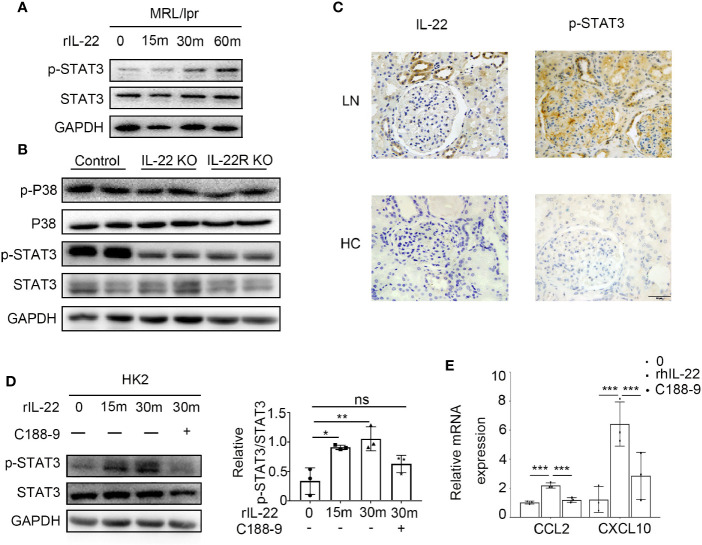
IL-22 promote the expression of CCL2, CXCL10 by activating the STAT3 pathway. **(A)** The phosphorylation level of STAT3 in primary kidney epithelial cells from control mice treated with or without rIL-22 (100 ng/ml) by western blotting. **(B)** Western blotting showed phosphorylation level of STAT3 and p38 in kidneys from 24-weeks-old control, IL-22 KO and IL-22R KO mice. **(C)** Representative photos of P-STAT3 and IL-22 in lupus nephritis patients and healthy control (HC) by immunochemistry (scale bar, 50 μm). **(D)** The phosphorylation level of STAT3 in HK2 cells treated with rIL-22 (100 ng/ml) by western blotting. Pretreating with or without C188-9 (STAT3 pathway inhibitor), HK-2 were treated with recombinant IL-22 for 0, 15, 30 min. **(E)** Following 24 h pre-treatment with or without C188-9, the relatively mRNA expression of chemokines in HK2 cells after treated with rIL-22 for 0 or 30 min. Values are normalized to GAPDH transcripts and expressed as relative ratio. Data are representative of three independent experiments. T-test was used for comparison between two groups (*P < 0.05, **P < 0.01, ***P < 0.001).

By immunoblotting, we detected the signaling pathway activated by IL-22/IL-22R in kidney epithelial cells of lupus-prone Mice. It was shown that the level of phosphorylated STAT3 significantly increased when primary control mouse kidney epithelial cells was treated with rIL-22 (**Figure 7A**). *In vivo*, the phosphorylation of STAT3 was much higher in the kidney of control mice at 24 -weeks age than that of IL-22 KO or IL-22R KO mice. But there was no difference in p38 between IL-22 KO or IL-22R KO mice and control mice (**Figure 7B**).

Immunochemistry results indicated increased levels of phosphorylation STAT3 and IL-22 in the kidney of LN patients when compared with healthy controls (**Figure 6C**). In human kidney cell line (HK2) cells, we observed similar results with mouse kidney epithelial cells. Moreover, when cells were treated with rIL-22 for 30 min following 24 h pre-treatment with STAT3 pathway inhibitor C188-9, the relatively mRNA expression of CCL2 and CXCL10 were significantly decreased in HK2 cells (**Figures 6D, E**).

## Discussion

In our study, we found that IL-22 was mainly secreted by ILC3 in MRL/lpr mice, and deleting IL-22 or IL-22R decreased the systemic illness and lupus nephritis severity in lupus-prone mice. We also found that deleting IL-22 or IL-22R downregulated CCL2 and CXCL10 expression, and decreased the filtration of macrophage into the kidney of lupus-prone mice. The phosphorylation of STAT3 was inhibited in the kidney of IL-22 or IL-22R KO mice*. In vitro* studies showed that primary kidney epithelial cells from control mouse stimulated with recombinant IL-22 (rIL-22) expressed higher levels of CCL2, CXCL10, and phosphorylated STAT3, and their supernatant recruited more macrophages. These results revealed that IL-22 may be involved in the pathogenesis of LN through activating STAT3 signaling and promoting CCL2 and CXCL10 expression and the infiltration of macrophage in kidney.

To date, there has been no consensus about which cells has been responsible for the production of IL-22 in SLE. Most studies have demonstrated that IL-22 in peripheral blood of SLE patients was expressed by effector CD4^+^ T cells (15). In the present study, we found that among IL-22^+^ cells, IL-22-producing ILC3s was nearly 60% in renal tissue of MRL/lpr mice, suggesting that IL-22 was mainly produced by ILC3s in LN. ILCs have emerged as important effector cells of the innate immune system and can secrete numerous cytokines, such as IFN-γ, IL-13, IL-22, while using specific mechanisms that directly kill target cells (16). Studies detected abnormal ILCs in a variety of autoimmune diseases, which led to abnormal immune activation and chronic inflammatory diseases (17). In humans and mice models of RA, the circulating ILC2s subset is crucial for the resolution of disease activity (18). As for SLE, one report that circulating ILC1s was increased in patients with SLE compared with HC (19), others report that increased ILC1s/ILC3s in Peripheral blood of SLE patients was correlated with nephritis and disease activity (20), and increased ILC3s may be related to the presence of a type I IFN signature (21). However, no studies so far have assessed ILCs in affected tissues of patients with SLE. We report that IL-22 was produced mainly by ILC3s in renal tissue of LN, suggesting ILC3s might contribute to renal tissue injury in LN. Additionally, we found the proportion of ILC3s co-expressing IL-22 and CCR6 (IL-17 producing ILC3s) in kidney of MRL/lpr mice was elevated with the development of LN, indicating that ILC3s simultaneously produced IL-17, which is acknowledged as pro-inflammatory cytokine for LN, and promote the development of LN. Therefore, studying local ILCs will add to the understanding of the role of ILCs in SLE immunopathology.

In the knockout experiment, we found that IL-22 KO or IL-22R KO MRL/lpr mice survived longer than control mice, and had less skin lesions, proteinuria and renal function and pathological impairment that is consistent with what we have shown in the past with anti-IL-22 mAb treatment for MRL/lpr mice (11). These results confirmed the pathogenic role of IL-22 in LN. Meanwhile, we found that IL-22 KO or IL-22R KO MRL/lpr mice had milder lymphadenopathy and splenomegaly, which suggesting IL-22 or IL-22R deficiency may also reduce systemic immune response. In LN, deposits of immune complex (ICs) in the glomeruli are regularly found and these activate complements in the kidneys, which results in decreased levels of serum C3 (22), and serum C3 levels and C3b deposition in the kidneys is are a good markers for LN (23). In the present study, we found indeed, in lupus-prone mice, IL-22 or IL-22R deficiency decreased production levels of serum ds-DNA autoantibodies and the levels of serum IgG, and increased the levels of serum C3. And decreased depositions of IgG and C3 in kidneys of IL-22 or IL-22R KO mice, suggesting that IL-22 may promote the production of autoantibodies and ICs. Though IL-22 is produced by immune cells, it cannot directly act on immune cells including B cells (7). Therefore, we speculated that IL-22 may upregulate immuno-inflammatory responses, resulting in immune dysregulation, which promote B cells to produce autoantibodies, ICs formation and C3 consumption.

Indeed, in lupus-prone mice, IL-22 or IL-22R deficiency decreased production of ds-DNA autoantibodies and the levels of serum IgG, and increased the levels of serum C3. The amount of renal IgG and C3 depositions in kidneys of IL-22 or IL-22R KO mice was also decreased. The findings pointed to the fact that in the absence of IL-22 or IL-22R, lupus-prone mice do not exhibit some of the readily recognizable features of immune dysregulation characteristic of lupus. Thus, IL-22 KO or IL-22R KO MRL mice may decrease the pathologic glomerular IC and C3 accumulation and in the kidneys. In other words, abrogation of the IL-22/IL-22R signaling may affect the production of Ig and, more importantly, of pathogenic autoantibodies.

A large number of infiltrating inflammatory cells, including T cells, B cells, neutrophils and macrophages are present in renal tissue of LN, following the formation and/or deposition of ICs in the kidney (24). As demonstrated above, control mice had much more cells per glomerulus than IL-22 or IL-22R KO mice. By flow cytometry, we further found fewer infiltrating macrophages into the kidney of IL-22 or IL-22R KO MRL/lpr mice than MRL/lpr mice, which confirmed by immunohistochemical analyses, but there was no significant difference in infiltrating T cells, B cells and neutrophils between IL-22 or IL-22R KO MRL/lpr mice and MRL/lpr mice, suggesting IL-22 may mainly affect the infiltration of Macrophages. Macrophages are believed to contribute to the pathogenesis of LN. In human and murine LN, renal macrophages infiltration is associated with active disease and poor outcomes (25, 26). Our study revealed that IL-22 may contribute to the pathogenesis of LN by promoting the infiltration of macrophages into the kidney.

There is no definitive evidence that IL-22 has capacity to act on hematopoietic cells including macrophages, though one report identified that incubation of DCs with rIL-22 augmented its ability to promote allergic inflammation (27). But binding IL-22 to IL-22R can induce nonhematopoietic cells to produce a variety of inflammatory mediators including cytokines, chemokines, etc (28). By RNA-Seq, we found that gene expression of local chemokines in the kidney of lupus-prone mice was altered, and qRT-PCR further confirmed down-regulated expression of CXCL1, CCL2, and CXCL10 in the kidney of IL-22 or IL-22R KO MRL/lpr mice compared with MRL/lpr mice. Among these chemokines, CCL2 and CXCL10 are demonstrated to play a critical role in promoting the recruitment of monocytes/macrophages into the kidney in SLE (29–31). Though little is known whether CXCL1 recruit inflammatory cells to the kidney in SLE, recent report showed that CXCL1 signaling can induce the infiltration of monocytes in heart and arteriogenic collaterals (32, 33). Our experiments on primary mouse kidney epithelial cells further confirmed the hypothesis. Our study demonstrated that primary kidney epithelial cells from MRL/lpr mouse stimulated with rIL-22 expressed higher levels of CCL2, CXCL10, and their supernatant recruited more macrophages. These findings indicated that IL-22 may upregulate the expression of CCL2 and CXCL10 and then promote the infiltration of macrophages into the kidney in the pathogenesis of LN.

In the present study, *in vivo*, lower phosphorylation of STAT3 in the kidney of IL-22 or IL-22R KO MRL/lpr mice was found than that in MRL/lpr mice. *In vitro*, with rIL-22 stimulation, levels of phosphorylation of STAT3 in primary kidney epithelial cells and HK2 cells were significantly increased, suggesting that STAT3 signaling pathway may be activated by IL-22-IL-22R in both human and murine kidney cells. Meanwhile, utilizing HK2 cells revealed that following the phosphorylation of STAT3 activated by rIL-22, levels of CCL2 and CXCL10 was highly expressed, and in turn, with inhibition of STAT3 pathway, levels of CCL2 and CXCL10 was decreased. These results phenocopied that of IL-22/IL-22R deficiency mice during LN as above demonstrated, implicating a requirement for STAT3 *in vivo* IL-22-mediating signaling.

In conclusion, we found that IL-22 secreted mainly by local ILC3s acted on kidney cells to enhance STAT3 phosphorylation and over-expression of CCL2 and CXCL10, thus promoting infiltration of macrophages into the kidney and then aggravating LN, and that IL-22 or IL-22R KO delayed the disease development of lupus-prone mice. These data revealed that IL-22 may play a pathogenic role in LN, and therefore, blockade of IL-22 or IL-22R may represent an attractive new strategy for treatment of LN.

## Data Availability Statement

The datasets presented in this study can be found in online repositories. The names of the repository/repositories and accession number(s) can be found below: NCBI SRA, BioProject PRJNA648341.

## Ethics Statement

The studies involving human participants were reviewed and approved by the ethics committee of the Second Affiliated Hospital, College of Medicine, Zhejiang University. Written informed consent for participation was not required for this study in accordance with the national legislation and the institutional requirements. The animal study was reviewed and approved by the ethics committee of the Second Affiliated Hospital, College of Medicine, Zhejiang University.

## Author Contributions

Research studies were designed by LH, XY, and QW. Experiments were performed and data acquired by LH, JH, LC, and YZ. Data were analyzed by LH, JH, LC, and YZ. LH and XY wrote the manuscript. All authors contributed to the article and approved the submitted version.

## Funding

This work was supported by grants from the National Natural Science Foundation of China (no. 81571578 to XY and no. 81930041 to QW) and the Natural Science Foundation of Zhejiang Province (no: LY20H100006 to XY).

## Conflict of Interest

The authors declare that the research was conducted in the absence of any commercial or financial relationships that could be construed as a potential conflict of interest.

## Supplementary Material

Supplementary Figure 1 The number of IL-22+ T cells in kidney. The absolute number of IL-22^+^ T cells (IL-22^+^CD3^+^) in the kidneys from 6-weeks or 24-weeks-old MRL/Fas Mice were analyzed by flow cytometry. Data were expressed as mean ± SD, and are representative of three independent experiments. T-test was used for comparison between two groups (**P*<0.05, ***P*<0.01, ****P*<0.001).

Supplementary Figure 2 Isotype control for Lin in kidney. The isotype control for Lin in the kidneys from 24W MRL/Fas Mice by flow cytometry.

## References

[1] Rahman A Isenberg DA. Systemic lupus erythematosus. N Engl J Med (2008) 358(9):929–39. 10.1056/NEJMra071297 18305268

[2] Kronbichler A Brezina B Gauckler P Quintana LF Jayne DRW. Refractory lupus nephritis: When, why and how to treat. Autoimmun Rev (2019) 18(5):510–8. 10.1016/j.autrev.2019.03.004 30844548

[3] Almaani S Meara A Rovin BH. Update on Lupus Nephritis. Clin J Am Soc Nephrol (2017) 12(5):825–35. 10.2215/CJN.05780616 PMC5477208 27821390

[4] Hanly JG O’Keeffe AG Su L Urowitz MB Romero-Diaz J Gordon C. The frequency and outcome of lupus nephritis: results from an international inception cohort study. Rheumatol (Oxford) (2016) 55(2):252–62. 10.1093/rheumatology/kev311 PMC4939728 26342222

[5] Shabgah AG Navashenaq JG Shabgah OG Mohammadi H Sahebkar A. Interleukin-22 in human inflammatory diseases and viral infections. Autoimmun Rev (2017) 16(12):1209–18. 10.1016/j.autrev.2017.10.004 29037907

[6] Wolk K Kunz S Witte E Friedrich M Asadullah K Sabat R. IL-22 increases the innate immunity of tissues. Immunity (2004) 21(2):241–54. 10.1016/j.immuni.2004.07.007 15308104

[7] Dudakov JA Hanash AM van den Brink MR. Interleukin-22: immunobiology and pathology. Annu Rev Immunol (2015) 33:747–85. 10.1146/annurev-immunol-032414-112123 PMC4407497 25706098

[8] Perriard G Mathias A Enz L Canales M Schluep M Gentner M. Interleukin-22 is increased in multiple sclerosis patients and targets astrocytes. J Neuroinflamm (2015) 12:119. 10.1186/s12974-015-0335-3 PMC4480507 26077779

[9] Ikeuchi H Kuroiwa T Hiramatsu N Kaneko Y Hiromura K Ueki K. Expression of interleukin-22 in rheumatoid arthritis: potential role as a proinflammatory cytokine. Arthritis Rheum (2005) 52(4):1037–46. 10.1002/art.20965 15818686

[10] Boniface K Guignouard E Pedretti N Garcia M Delwail A Bernard FX. A role for T cell-derived interleukin 22 in psoriatic skin inflammation. Clin Exp Immunol (2007) 150(3):407–15. 10.1111/j.1365-2249.2007.03511.x PMC2219373 17900301

[11] Yang X Weng Q Hu L Yang L Wang X Xiang X. Increased interleukin-22 levels in lupus nephritis and its associated with disease severity: a study in both patients and lupus-like mice model. Clin Exp Rheumatol (2019) 37(3):400–7. 30299250

[12] Yang X Gao Y Wang H Zhao X Gong X Wang Q. Increased urinary interleukin 22 binding protein levels correlate with lupus nephritis activity. J Rheumatol (2014) 41(9):1793–800. 10.3899/jrheum.131292 25086075

[13] Hochberg MC. Updating the American College of Rheumatology revised criteria for the classification of systemic lupus erythematosus. Arthritis Rheum (1997) 40(9):1725. 10.1002/art.1780400928 9324032

[14] Chong Z Bao C He J Chen T Zhong L Li G. E3 ligase FBXW7 aggravates TMPD-induced systemic lupus erythematosus by promoting cell apoptosis. Cell Mol Immunol (2018) 15(12):1057–70. 10.1038/s41423-018-0167-z PMC6269436 30275535

[15] Duhen T Geiger R Jarrossay D Lanzavecchia A Sallusto F. Production of interleukin 22 but not interleukin 17 by a subset of human skin-homing memory T cells. Nat Immunol (2009) 10(8):857–63. 10.1038/ni.1767 19578369

[16] Artis D Spits H. The biology of innate lymphoid cells. Nature (2015) 517(7534):293–301. 10.1038/nature14189 25592534

[17] Xiong T Turner J-E. Innate lymphoid cells in autoimmunity and chronic inflammatory diseases. Semin Immunopathol (2018) 40(4):393–406. 10.1007/s00281-018-0670-4 29568972

[18] Rauber S Luber M Weber S Maul L Soare A Wohlfahrt T. Resolution of inflammation by interleukin-9-producing type 2 innate lymphoid cells. Nat Med (2017) 23(8):938–44. 10.1038/nm.4373 PMC5575995 28714991

[19] Shikhagaie M Germar K Bal S Ros X Spits H. Innate lymphoid cells in autoimmunity: emerging regulators in rheumatic diseases. Nat Rev Rheumatol (2017) 13(3):164–73. 10.1038/nrrheum.2016.218 28148916

[20] Hou M Liu S. Innate lymphoid cells are increased in systemic lupus erythematosus. Clin Exp Rheumatol (2019) 37(4):676–9. 30789153

[21] Blokland SLM van den Hoogen LL Leijten EFA Hartgring SAY Fritsch R Kruize AA. Increased expression of Fas on group 2 and 3 innate lymphoid cells is associated with an interferon signature in systemic lupus erythematosus and Sjögren’s syndrome. Rheumatol (Oxford England) (2019) 58(10):1740–5. 10.1093/rheumatology/kez116 31220315

[22] Aringer M Costenbader K Daikh D Brinks R Mosca M Ramsey-Goldman R. 2019 European League Against Rheumatism/American College of Rheumatology classification criteria for systemic lupus erythematosus. Ann Rheum Dis (2019) 78(9):1151–9. 10.1136/annrheumdis-2019-216700 31383717

[23] Leffler J Bengtsson AA Blom AM. The complement system in systemic lupus erythematosus: an update. Ann rheumatic Dis (2014) 73(9):1601–6. 10.1136/annrheumdis-2014-205287 24845390

[24] Flores-Mendoza G Sansón SP Rodríguez-Castro S Crispín JC Rosetti F. Mechanisms of Tissue Injury in Lupus Nephritis. Trends Mol Med (2018) 24(4):364–78. 10.1016/j.molmed.2018.02.003 29526595

[25] Li J Liu C-H Xu D-L Gao B. Significance of CD163-Positive Macrophages in Proliferative Glomerulonephritis. Am J Med Sci (2015) 350(5):387–92. 10.1097/MAJ.0000000000000569 26379042

[26] Maria NI Davidson A. Renal Macrophages and Dendritic Cells in SLE Nephritis. Curr Rheumatol Rep (2017) 19(12):81. 10.1007/s11926-017-0708-y 29119288

[27] Schnyder B Lima C Schnyder-Candrian S. Interleukin-22 is a negative regulator of the allergic response. Cytokine (2010) 50(2):220–7. 10.1016/j.cyto.2010.02.003 20194033

[28] Aujla SJ Chan YR Zheng M Fei M Askew DJ Pociask DA. IL-22 mediates mucosal host defense against Gram-negative bacterial pneumonia. Nat Med (2008) 14(3):275–81. 10.1038/nm1710 PMC2901867 18264110

[29] Chalmers SA Chitu V Ramanujam M Putterman C. Therapeutic targeting of macrophages in lupus nephritis. Discovery Med (2015) 20(108):43–9. 26321086

[30] Antonelli A Ferrari SM Giuggioli D Ferrannini E Ferri C Fallahi P. Chemokine (C-X-C motif) ligand (CXCL)10 in autoimmune diseases. Autoimmun Rev (2014) 13(3):272–80. 10.1016/j.autrev.2013.10.010 24189283

[31] Lit LCW Wong CK Tam LS Li EKM Lam CWK. Raised plasma concentration and ex vivo production of inflammatory chemokines in patients with systemic lupus erythematosus. Ann rheumatic Dis (2006) 65(2):209–15. 10.1136/ard.2005.038315 PMC1798029 15975968

[32] Wang L Zhang Y-L Lin Q-Y Liu Y Guan X-M Ma X-L. CXCL1-CXCR2 axis mediates angiotensin II-induced cardiac hypertrophy and remodelling through regulation of monocyte infiltration. Eur Heart J (2018) 39(20):1818–31. 10.1093/eurheartj/ehy085 29514257

[33] Vries MHM Wagenaar A Verbruggen SEL Molin DGM Dijkgraaf I Hackeng TH. CXCL1 promotes arteriogenesis through enhanced monocyte recruitment into the peri-collateral space. Angiogenesis (2015) 18(2):163–71. 10.1007/s10456-014-9454-1 25490937

