# Supplementary figures and images for: Interleukin-22 From Type 3 Innate Lymphoid Cells Aggravates Lupus Nephritis by Promoting Macrophage Infiltration in Lupus-Prone Mice
Source: Front Immunol. 2021 Feb 26;12:584414. doi: 10.3389/fimmu.2021.584414 (PMC7953152; doi:10.3389/fimmu.2021.584414)

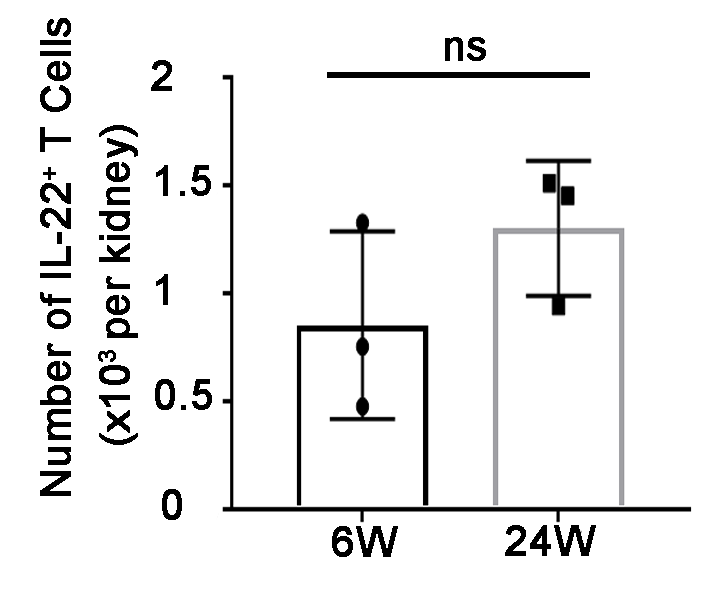

Supplement: Supplementary Figure 1 — The number of IL-22+ T cells in kidney. The absolute number of IL-22+ T cells (IL-22+CD3+) in the kidneys from 6-weeks or 24-weeks-old MRL/Fas Mice were analyzed by flow cytometry. Data were expressed as mean ± SD, and are representative of three independent experiments. T-test was used for comparison between two groups (*P<0.05, **P<0.01, ***P<0.001). [file Image_1.tif]

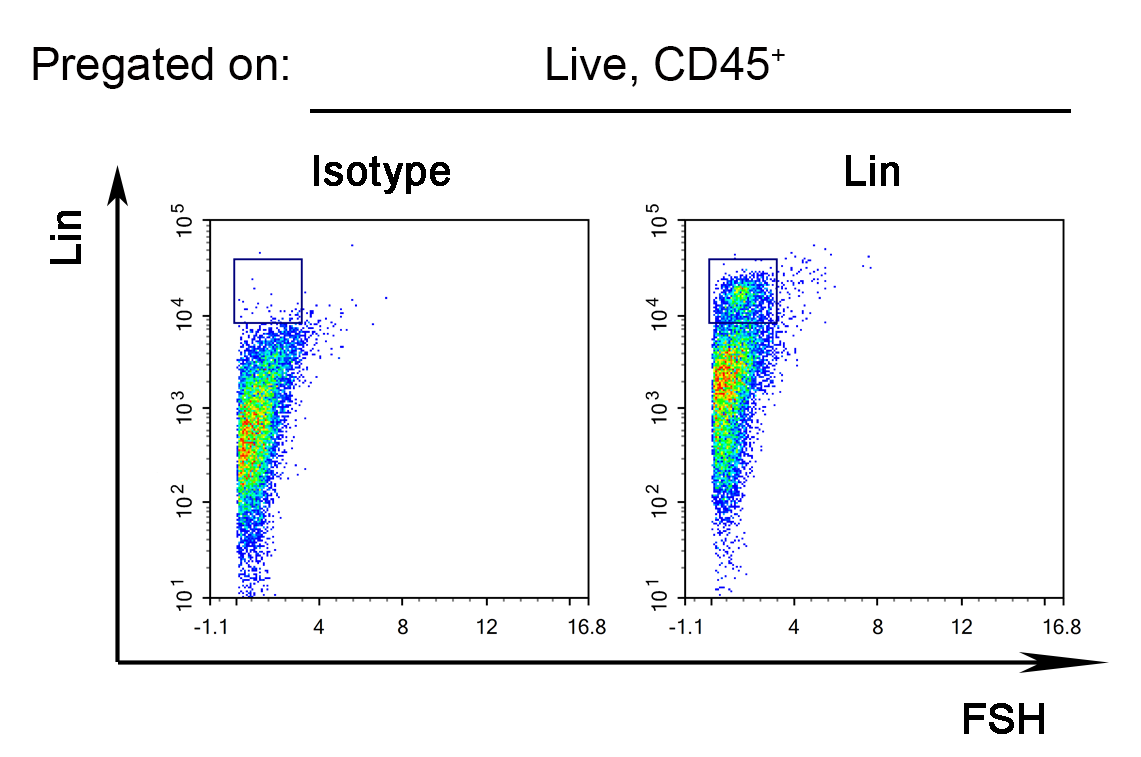

Supplement: Supplementary Figure 2 — Isotype control for Lin in kidney. The isotype control for Lin in the kidneys from 24W MRL/Fas Mice by flow cytometry. [file Image_2.tif]
