# Supplementary material for: Interleukin-22 From Type 3 Innate Lymphoid Cells Aggravates Lupus Nephritis by Promoting Macrophage Infiltration in Lupus-Prone Mice
Source: Front Immunol. 2021 Feb 26;12:584414. doi: 10.3389/fimmu.2021.584414 (PMC7953152; doi:10.3389/fimmu.2021.584414)
Supplement: Supplementary file 3 [file Table_1.docx]

Supplementary Table 1: List of primers used in this study.

| Table 1. Primers for RT-PCR analysis | | |
| --- | --- | --- |
| Genes | Forward primer (5′-3′) | Reverse primer (5′-3′) |
| mCCL2 | TTAAAAACCTGGATCGGAACCAA | GCATTAGCTTCAGATTTACGGGT |
| mCXCL1 | ACCGAAGTCATAGCCACACTC | CTCCGTTACTTGGGGACACC |
| mCXCL10 | CCACGTGTTGAGATCATTGCC | GAGGCTCTCTGCTGTCCATC |
| mActin | AGTGTGACGTTGACATCCGT | GCAGCTCAGTAACAGTCCGC |
| hCCL2 | AGAGGCTGAGACTAACCCAGA | TTTCATGCTGGAGGCGAGAG |
| hCXCL1 | AGCTCTTCCGCTCCTCTCA | CACGGACGCTCCTGCTG |
| hCXCL10 | CCACGTGTTGAGATCATTGCT | TGCATCGATTTTGCTCCCCT |
| hGAPDH | TGTGGGCATCAATGGATTTGG | ACACCATGTATTCCGGGTCAAT |
