# Supplementary material for: Interleukin-22 From Type 3 Innate Lymphoid Cells Aggravates Lupus Nephritis by Promoting Macrophage Infiltration in Lupus-Prone Mice
Source: Front Immunol. 2021 Feb 26;12:584414. doi: 10.3389/fimmu.2021.584414 (PMC7953152; doi:10.3389/fimmu.2021.584414)
Supplement: Supplementary file 4 [file Table_2.docx]

Supplementary Table 2: List of antibodies used in this study.

| Table 1. Antibodies for flow cytometry analysis | |
| --- | --- |
| Peptide/Protein Target | Supplier |
| FITC anti-B220 | BD |
| Percp/Cy5.5 anti-CCR6 | Biolegend |
| PE anti-CD11b | Biolegend |
| FITC anti-CD127 | Biolegend |
| anti-CD16/32 | Biolegend |
| APC anti-CD3 | Biolegend |
| APC/Cy7 anti-CD45 | Biolegend |
| BV421 anti-F4/80 | Biolegend |
| PB anti-Lineage | Biolegend |
| FITC anti-Ly6G | BD |
| APC anti-RORγt | Biolegend |
